# Trueness of Extraoral Digital Impressions for Full-Arch Implant Impressions—In Vitro Study

**DOI:** 10.3390/ma17122932

**Published:** 2024-06-15

**Authors:** Manuel António Sampaio-Fernandes, Ricardo Pinto, Paulo Rocha Almeida, Maria Margarida Sampaio-Fernandes, Duarte Marques, Maria Helena Figueiral

**Affiliations:** 1 Faculdade de Medicina Dentária, Universidade do Porto, 4200-393 Oporto, Portugal; prochalmeida@gmail.com; 2 Faculdade de Medicina Dentária, Universidade de Lisboa, 1600-277 Lisbon, Portugal; ricardojpinto@campus.ul.pt; 3 Faculdade de Medicina Dentária and INEGI researcher, Universidade do Porto, 4200-393 Oporto, Portugal; margaridasampaiofernandes@gmail.com (M.M.S.-F.); hfigueiral@gmail.com (M.H.F.)

**Keywords:** cast scan, digital implant impression, extraoral scanner, full-arch rehabilitation, impression scan, impression trueness, technologies

## Abstract

Direct scanning of silicone impressions is a valid technique. However, studies in implant-supported rehabilitations are lacking. This in vitro study aims to compare the trueness of impressions obtained with two types of silicone and their corresponding stone casts, using two laboratory scanners in a full-arch implant rehabilitation. A master cast with six dental implants was scanned with a 12-megapixel scanner to obtain a digital master cast. Ten implant impressions were made using two silicones (Zhermack and Coltene) with the open-tray technique. The impressions and stone casts were scanned by two extraoral scanners (Identica T500, Medit; and S600 ARTI, Zirkonzhan). Trueness was assessed by comparing linear and angular distances in digital casts with the master cast. A *p* < 0.05 significance level was considered. The results showed that for the linear measurements, 72% were higher than the master cast measurements, and no consistent pattern was observed in the angular measurements. The greatest deviations were detected between the most posterior implants, with mean values ranging between 173 and 314 µm. No significant differences were found between scanners. However, differences were observed in the distances between silicones (46.7%) and between impressions and stone casts (73.3%). This work demonstrates that the direct scanning of silicone impressions yields results comparable to those obtained from scanning gypsum casts in full-arch implant-supported rehabilitation.

## 1. Introduction

Implant-supported prostheses are viable alternatives to conventional prostheses for patients with tooth loss, offering long-term stability and comfort [1,2].

The fit of a fixed dental prosthesis on implants is essential for the success of long-term rehabilitation [3,4,5]. Thus, the accuracy of the impression technique and the working cast are essential requirements to achieve a passive fit of the prosthesis [3,6,7]. A restoration is considered to have a passive fit when no static loads are generated within the prosthetic system or in the surrounding bone tissue [8]. Prosthetic misfit increases the incidence of mechanical complications such as occlusal discrepancies and loosening or fracture of prosthetic and implant components and enhances the accumulation of bacterial plaque, which affect the surrounding tissues of the implants [3,8]. It can also increase stress on the surrounding bone, resulting in marginal bone loss and potentially leading to implant failure [2]. An osseointegrated implant has extremely limited ability to move within the 10 µm range, whereas a natural tooth can move up to 100 µm in the periodontal ligament. Therefore, the fit of implant-supported prostheses is more critical than in tooth-supported prostheses [9]. Misfitted implant-supported prostheses can negatively affect the long-term success rate, especially in immediate loading procedures [3,5].

The accuracy of the impression taken is an essential aspect. Accuracy is defined by trueness and precision. Trueness is defined as “the proximity between the average value obtained from a large series of test results and a reference value”, and precision is defined as the proximity between independent test results obtained under defined conditions [10,11].

Digital impressions with an intraoral scanner have been described as being as accurate or even superior to conventional impressions for single crowns or fixed prostheses limited to one quadrant [10,12]. However, in completely edentulous arches, the accuracy of intraoral scanners decreases significantly, as larger parts of the arch are captured and a greater number of images must be superimposed, leading to accumulate inaccuracies and increased global distortion [12,13,14]. Several studies have confirmed that the greater the distance between implants, the lower the accuracy of intraoral scans [13,14]. In contrast, the use of extraoral scanners does not exhibit this deviation pattern, as they capture several sections of the casts simultaneously, minimizing potential errors [12,15,16]. Furthermore, this method avoids the need to create the solid stone model, thus eliminating potential errors and distortions [17].

Obtaining an accurate transfer of implant position and angulation is a crucial factor in achieving a satisfactory prosthesis with a precision fit [8,9,18,19].

The objectives of this in vitro study are to evaluate the trueness of digital implant impressions obtained with two types of silicone and their corresponding stone casts, using two laboratory scanners in a full-arch implant rehabilitation. The null hypotheses are that the (1) scanner, (2) silicone, and (3) pouring dental cast do not affect the trueness of the impressions.

## 2. Materials and Methods

**Figure 1:**
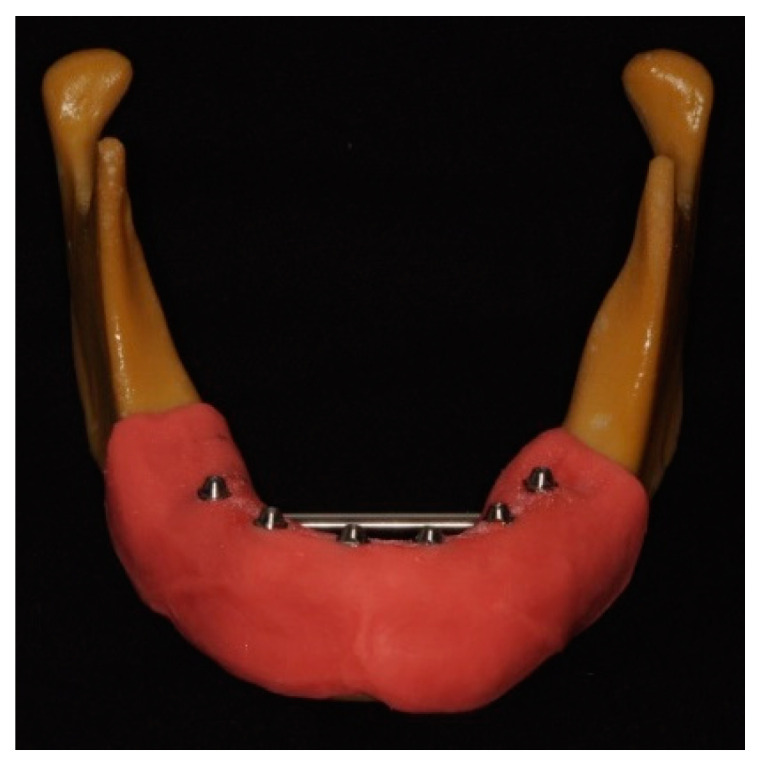
Master cast used in this study. Distribution of implants: M1r, right first molar; P1r, right first premolar; I2r, right lateral incisor; I2l, left lateral incisor; P1l, left first premolar; M1l, right first molar.

Six Straumann bone-level 4.1 implants with an internal hexagonal connection (Institut Straumann AG, Basel, Switzerland) and corresponding screw-retained abutments (SRA) (Institut Straumann AG, Basel, Switzerland) were placed in an artificial mandible with artificial gingiva, simulating a clinical situation (Figure 1). The master cast was scanned with a 12-megapixel reference scanner (Gom Atos Compact Scan 12 M, GOM Metrology, Braunschweig, Germany), saved as an STL file and considered the digital master cast.

**Figure 2:**
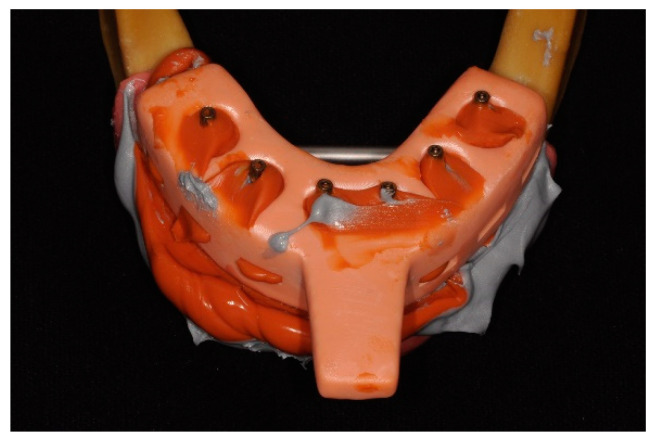
Silicone impression with open-tray technique.

Based on the master cast, impressions were taken with two silicones with an open tray and splinted copings using the double-mix technique: Group A—10 impressions with Light Body Type III, Heavy Body Type I, Hydrorise Implant, Zhermack, Rovigo, Italy; Group B—10 impressions with Light Body Type III, Heavy Body Type I, Coltene, Altstätten, Switzerland. The trays were made using 3D printing and pre-coated with silicone adhesive (Coltene, Altstätten, Switzerland) before taking the impressions. The copings were screwed at 15 Ncm into the SRAs and splinted with methylmethacrylate. The splint was sectioned 24 h after polymerization; then, more resin was added in small amounts. After polymerization, the copings were unscrewed, and the impression was removed from the master cast (Figure 2). Subsequently, the leaking impression material and the buccal surface of the impression were cut with a scalpel to allow light from the scanner to enter.

**Figure 3:**
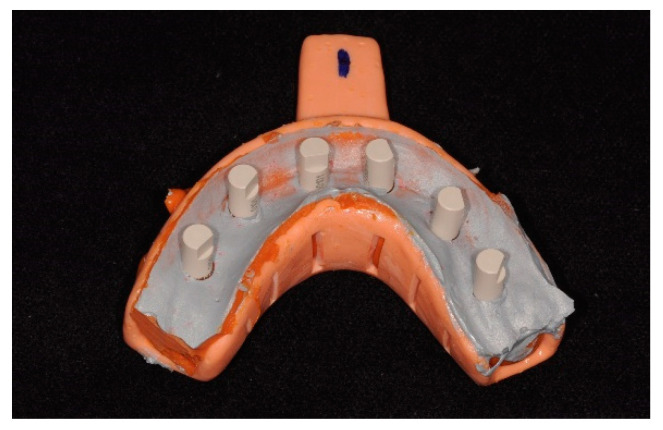
Silicone impression with ScanAnalogs.

For the digitalization of the master cast impressions with extraoral scanners, ScanAnalogs (dynamic abutment solutions) were screwed into the copings, and impressions were sprayed with a uniform minimum thickness coating of TiO_2_ particles according to ISO-12836 [20] (Figure 3). The impressions were digitalized with two laboratory scanners: a blue-LED scanner (Identica T500; Medit, Seoul, Republic of Korea) and a structured-light optical scanner (S600 ARTI; Zirkonzahn, South Tyrol, Italy). The images obtained were saved in STL files. Both conventional and digital impressions were taken by a single operator experienced in the abovementioned techniques.

Prior to gypsum pouring, the ScanAnalogs were removed, and the multiunit analogs were screwed into the transfers; subsequently, the impressions were cleaned and dried. Then, artificial gingiva was placed on the impressions (Gingifast elastic; Zhermack, Rovigo, Italy). Type IV plaster (Elite Rock; Zhermack, Rovigo, Italy) was used for casting, according to the manufacturer’s recommendations. The impressions were poured under constant vibration, and after 2 h the impression/cast were separated by unscrewing the transfers.

For digitalization with extraoral scanners, ScanBodies were placed, and the stone casts were sprayed with TiO_2_ particles. The scanning was performed with the two described scanners and saved in STL files. After digitalization, eight digital cast study groups were obtained (Table 1).

The scans obtained were edited in Exocad for the placement of digital replicas corresponding to the ScanBodies and ScanAnalogs used. After this process, files were exported in STL with the virtual position of each implant.

**Figure 4:**
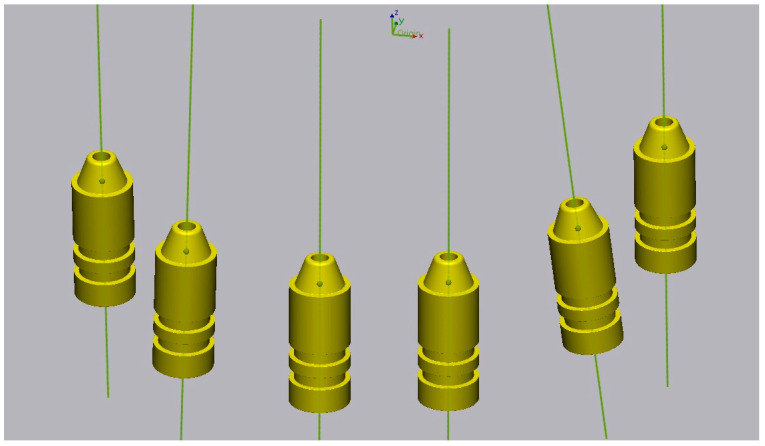
Midpoint and vector of each implant.

The zero method was used: for each virtual cast, the midpoint and the vector corresponding to the virtual position of the implants were defined (Figure 4). Finally, the linear measurements between the midpoints and the angular measurements between the vectors were compared between the master cast and the virtual casts created using 3D analysis software (Geomagic Control X 2022.0.1; 3D Systems, Rock Hill, SC, USA). Thirty measurements (15 linear and 15 angular) were performed on each virtual cast, for a total of 2400 measurements. The measurements were then compared with the master cast measurements using absolute values in a statistical analysis program.

The data obtained are presented as median and IQR values and mean ± confidence interval (CI) at 95% for the different locations. The normality of distribution was tested by the Shapiro–Wilk test and the equality of variance by the Levene test. The non-parametric Mann–Whitney U test was used on data with non-normal distribution and the *T*-Test was used on data with normal distribution to compare discrepancies between groups at different locations (α = 0.05). Results were considered statistically significant at *p* < 0.05. Statistical analysis was conducted using the software SPSS 25.0 (SPSS Inc., Chicago, IL, USA). A statistical power analysis was performed to determine the sample size, with α = 0.05 and a power of 0.80with an equivalence study design.

## 3. Results

Results were calculated from 15 linear and 15 angular distances measured between six implants. A total of 2400 measurements were performed on 80 digital casts.

Of the 1200 linear measurements performed, 864 (72%) resulted in values higher than the measurements recorded on the master cast (Table 2). Evaluating the percentage of positive discrepancies between the most posterior implants of each quadrant, we verified that the percentage was 100% in all measurements carried out in all assessed casts, corresponding to an expansion of the cast in the posterior sector when compared to the master cast. Of the remaining linear distances analyzed, in less than 10% of the cases were discrepancies over 150 µm found, which is the value acceptable to avoid misfit frameworks [21].

There were no statistically significant differences between scanners for the distances and angles measured (Table 3 and Table 4). Between Coltene and Zhermack silicone, there were statistically significant differences in the measured distances between implants in different quadrants, except between the two most anterior implants and between right first molar (M1r) and left lateral incisor (I2l), with better results for Coltene. There were also statistically significant differences in 8 of the 15 measured angles, although with no apparent pattern. Between stone casts and impressions, statistically significant differences were detected, except for four of the five smaller inter-implant distances, with better results in the impression group. Also, between stone casts and impressions, there were statistically significant differences in only three angles, which were related to the same implant (I2l).

## 4. Discussion

This in vitro study did not detect statistically significant differences between the two extraoral scanners studied. However, there were statistically significant differences in some measured distances between the two silicones and between impressions and the corresponding stone casts. Thus, the first null hypothesis is accepted, while the second and third null hypotheses are rejected.

Accuracy is defined by precision and trueness. Precision represents the degree of reproducibility between repeated measurements, and trueness describes the closeness to real dimensions. Published studies have used linear distance measurements to investigate the trueness between casts [18]. The “zero method” described by Giménez et al. [22] was applied in this study, allowing the comparison of linear and angular distances in digital casts with those measured in the master cast.

In this investigation, we used two scanners with different technologies that were used in previous studies for extraoral scanning of conventional impressions. The Blue-LED scanner (Medit^®^) has a short wavelength and, consequently, high precision in deep areas. The structured white-light scanner (Zirkonzhan^®^) offers quick scanning and up to 10 μm accuracy according to the manufacturer. The extraoral digitalization eliminates the gypsum pouring step, consequently reducing time and laboratory costs [10,11].

The measurement methods used in this study are in accordance with the established and validated techniques for examining the accuracy of digital implant casts, particularly in cases of full-arch implant-supported prostheses where the degree of error is determined by the relative positions between implants [23].

Globally evaluating the results of linear distances, in most casts (72.1%), there was an expansion in relation to the master cast. Additionally, in the distances between the most posterior implants of each quadrant, the percentage of discrepancies greater than 150 µm ranged between 57.5% and 97.5% [21]. This is consistent with the literature, which notes an expansion of elastomers and stone casts, particularly towards the posterior region [24].

Studies have reported that conventional impression discrepancies are practically inevitable [3]. A study by Menini et al. [3] detected mean deviations of linear distances of −12 ± 26 µm and angular distances of 0.257 ± 0.242° in intraoral scans and deviations of linear distances of 60 ± 37 µm and angular distances of 0.503 ± 0.854° in stone cast scans with extraoral scanners. However, in the study described, positive and negative discrepancies were used, which may mask the overall results. In this investigation, discrepancies were calculated using absolute values. A study on impression techniques with six implants suggests that the biggest inaccuracies are more likely to be found between the most distant implants, regardless of the impression technique used [25]. In this study, the mean linear deviation was 72.89 µm. However, if we had not used absolute values, the mean would be 46.34 µm. The longer the distance measured, the greater the tendency to obtain larger deviations. In our study, the shortest distance (between implants I2r and I2l) was 34.23 µm, and the distance between the most posterior implants was 230.44 µm. According to the literature, linear distances greater than 150 µm can lead to framework misfit [21].

A study on inter-implant distances in impressions reported that there were no differences between the distances measured when using an extraoral scanner as opposed to intraoral scanners [16]. However, extraoral scanners capture an object from multiple angles over a wide area using a high-precision camera, with the object fixed at a certain distance. In contrast, intraoral scanners have a smaller camera size and capture data in a small area per scan, increasing error accumulation [16].

Regarding angular distances between implants, one study reported mean deviations of 0.27° in intraoral scans and 0.91° in stone cast scans [14]. In the present study, the mean was 0.32° for both stone casts and impressions.

Silicone has more resistance to deformation and a lower modulus of elasticity than polyethers (PEs), being a viable choice in cases of multiple implants, especially in non-parallel implants or those with deep internal connections [8]. Both silicones and PEs are reported as excellent materials for implant impressions [8,23]. Polyether siloxanes, recently introduced in prosthodontics, combine the properties of silicones and PEs [23]. Nevertheless, scientific evidence on the performance of these materials in implant impressions is currently scarce [23]. A study by Baig et al. [23] on implant impression materials detected discrepancies in linear distances between implants in stone casts of 106 µm for PEs and 103 µm for silicones. However, the study used a coordinate measuring machine (CMM) instead of the industrial scanner used in our investigation.

Regarding the impression technique, a systematic review from 2008 [19] reported that in the closed-tray technique, the copings do not return to the original position, which is the main source of error in this technique and may be amplified in situations with multiple implants. According to the literature, in situations with four or more implants, the open-tray technique is preferable to the closed-tray technique. The “snap-fit” technique, which combines advantages of both techniques, can be a reliable impression technique [19]. In this investigation, this technique was not used because it does not allow silicone digitalization with extraoral scanners. Given this scenario, we used the open-tray technique, which is the most appropriate in clinical situations with six or more implants.

The splinting of copings with self-curing acrylic resin prevents inter-implant movement during impression taking [3]. However, the inevitable contraction of the resin during polymerization is a distortion factor that can negatively affect the fit of implant-supported prostheses. Studies have reported contraction values of 7.9% after 24 h, with 80% of contraction occurring in the first 17 min, a rate proportional to the size of the ferrule [3]. This is particularly concerning in full-arch impressions. In this study, to minimize this effect, the splint was sectioned 24 h after polymerization; then, more resin was added in small amounts. It should be noted that this procedure does not represent an ideal solution in immediate loading treatments. Some studies report that splinting copings yields better results than non-splinting, although other studies did not find significant differences [6,23]. A systematic review from 2008 [19] noted that the non-splinting technique had better results for impressions, but the splinting technique is more accurate on stone casts.

Regarding ScanBodies, it has been shown that they can affect accuracy [6]. The use of the same ScanBody in several casts may increase discrepancies due to potential wear from successive tightening and loosening. To minimize this impact, ScanBodies were set at 10 Ncm and replaced every five uses.

This investigation did not consider the operator’s expertise and how it can impact the accuracy of dental impressions. According to the literature, the skill of the operator in using digital or conventional methods can significantly influence the results [17].

### Limitations

Possible sources of error in this study also include the use of spray to reduce reflectivity before scanning and alignment errors associated with measurements performed in Geomagic software. Operator errors can also occur, such as when screwing and unscrewing copings, ScanBodies, and ScanAnalogs, which must be done manually. To minimize this impact, all procedures were performed by the same calibrated operator with experience in the techniques used. Radiographs to control the position of the prosthetic components are recommended, but this was not done once the prosthetic components were completely radiolucent. Therefore, it is recommended that implant brands provide components with a radiopaque cervical neck.

The digital methodology used in this study allowed us to compare discrepancies between different impression acquisition methodologies in various clinical situations for full-arch implant-supported rehabilitations.

## 5. Conclusions

Within the limitations of this study, the following conclusions can be drawn:

1. There are no statistically significant differences in the trueness between the two scanners evaluated.
2. There are statistically significant differences in trueness between the two silicones evaluated in the vast majority of measured distances.
3. The direct scanning of silicone impressions by laboratory scanners presents comparable results to stone cast scanning.

The extraoral digitalization of impressions on edentulous dental arches with six implants proved to be a valid technique that allows the elimination of laboratory steps, including the gypsum pouring, without decreasing the trueness of the process.

## Figures and Tables

**Table 1:** Definition of the study groups.

Scanner	Material	Casts	Group
Medit	Coltene	Impressions	MCI
		Stone Casts	MCM
	Zhermack	Impressions	MZI
		Stone Casts	MZM
Zirkonzhan	Coltene	Impressions	ZCI
		Stone Casts	ZCM
	Zhermack	Impressions	ZZI
		Stone Casts	ZZM

**Table 2:** Number of positive cases and discrepancies > 150 µm for each linear distance.

Linear Distance	Positive Cases		Discrepancies > 150 µm	
	Frequency of Positive Cases	Frequency of Positive Cases (%)	Frequency of Discrepancies > 150 µm	Frequency of Discrepancies > 150 µm (%)
M1r-P1r	3	3.75%	0	0%
M1r-I1r	61	76.25%	0	0%
M1r-I1l	57	71.25%	6	7.5%
M1r-P1l	80	100%	54	67.5%
M1r-M1l	80	100%	78	97.5%
P1r-I1r	77	96.25%	1	1.25%
P1r-I1l	72	90%	8	1%
P1r-P1l	80	100%	28	35%
P1r-M1l	80	100%	46	57.5%
I1r-I1l	38	47.5%	6	7.5%
I1r-P1l	67	83.75%	7	8.75%
I1r-M1l	72	90%	6	7.5%
I1l-P1l	24	30%	0	0%
I1l-M1l	27	33.75%	0	0%
P1l-M1l	46	57.5%	0	0%
Total	864	72%	240	20%

M1r, right first molar; P1r, right first premolar; I2r, right lateral incisor; I2l, left lateral incisor; P1l, left first premolar; M1l, left first molar.

**Table 3:** Evaluation of linear trueness per group at each location (mean ± 95% CI) in µm.

	Medit Scanner				Zirkonzahn Scanner			
Linear Distance	Coltene Silicone	Coltene Stone Casts	Zhermack Silicone	Zhermack Stone Casts	Coltene Silicone	Coltene Stone Casts	Zhermack Silicone	Zhermack Stone Casts
M1r-P1r	56.02 [36.02; 72.02]	42.67 [18.82; 66.52]	53.76 [39.17; 67.76]	46.20 [25.89; 66.50]	69.78 [51.86; 87.70]	50.14 [28.60; 71.68]	57.98 [40.06; 75.90]	54.90 [25.20; 84.60]
M1r-I1r	20.02 [9.15; 30.89]	60.99 [38.69; 83.29]	14.05 [7.64; 20.46]	46.33 [31.12; 61.54]	16.39 [5.01; 27.77]	34.52 [13.47; 55.57]	14.89 [9.69; 20.09]	29.66 [21.92; 37.40]
M1r-I1l	25.51 [13.84; 37.18]	55.31 [34.66; 75.96]	47.59 [−0.79; 95.97]	85.88 [33.07; 138.69]	20.68 [13.44; 27.92]	39.39 [16.81; 61.97]	55.83 [5.68; 105.98]	73.50 [31.54; 115.46]
M1r-P1l	152.82 [125.05; 180.59]	191.89 [162.73; 221.05]	196.50 [163.65; 229.35]	231.26 [188.29; 274.23]	116.05 [95.90; 136.20]	165.06 [129.08; 201.04]	182.16 [149.44; 214.88]	228.22 [196.15; 260.29]
M1r-M1l	217.65 [180.38; 254.92]	278.83 [252.34; 305.32]	279.62 [257.02; 302.22]	305.69 [275.09; 336.29]	173.45 [145.77; 201.09]	250.39 [225.73; 275.05]	251.04 [221.26; 280.82]	313.68 [281.99; 345.37]
P1r-I1r	46.56 [32.15; 60.97]	75.81 [38.88; 112.74]	42.99 [34.95; 51.03]	79.78 [49.31; 110.25]	42.15 [27.21; 57.09]	71.56 [40.06; 103.06]	41.20 [26.05; 56.35]	67.21 [41.25; 93.17]
P1r-I1l	31.24 [17.13; 45.35]	52.71 [27.02; 78.40]	69.07 [5.82; 132.32]	117.11 [51.06; 183.16]	22.48 [10.43; 34.53]	61.08 [29.62; 92.54]	78.81 [5.13; 148.49]	98.70 [36.61; 160.79]
P1r-P1l	101.54 [78.59; 124.49]	124.16 [93.97; 154.35]	142.04 [96.04; 188.04]	206.71 [157.36; 256.06]	77.16 [56.22; 98.10]	127.27 [92.23; 162.31]	125.67 [75.25; 176.09]	188.00 [139.75; 236.25]
P1r-M1l	113.96 [88.04; 139.88]	166.86 [147.77; 185.95]	175.49 [141.83; 209.15]	230.09 [187.58; 272.60]	84.64 [58.12; 111.16]	162.35 [125.13; 199.57]	150.53 [111.55; 189.51]	220.84 [180.73; 260.95]
I1r-I1l	18.66 [8.63; 28.69]	39.52 [13.05; 65.99]	48.68 [−6.02;103.38]	54.99 [7.16; 102.82]	19.80 [10.44; 29.16]	26.66 [8.28; 45.04]	49.79 [−8.03; 107.61]	65.53 [22.34; 108.72]
I1r-P1l	28.09 [19.25; 36.93]	33.48 [11.85; 55.11]	68.10 [27.57; 108.63]	82.92 [40.65; 125.19]	19.11 [10.15; 28.07]	49.56 [35.27; 63.85]	55.70 [15.36; 96.04]	95.35 [55.49; 135.21]
I1r-M1l	24.94 [9.19; 40.69]	64.89 [34.25; 95.53]	86.66 [57.80; 115.52]	85.86 [53.18; 118.54]	123.88 [−108.47; 356.23]	66.13 [48.53; 83.73]	72.77 [44.20; 101.34]	113.74 [78.38; 149.10]
I1l-P1l	30.42 [20.45; 40.39]	29.00 [10.70; 47.30]	26.81 [15.04; 38.58]	22.08 [3.96; 40.20]	41.43 [28.29; 54.57]	24.35 [11.19; 37.51]	32.43 [15.87; 49.00]	44.45 [31.81; 57.09]
I1l-M1l	49.28 [31.22; 67.34]	30.64 [18.75; 42.53]	26.85 [12.63; 41.07]	20.08 [5.30; 34.85]	59.62 [39.13; 80.11]	13.24 [5.43; 21.05]	27.31 [11.93; 42.69]	36.88 [12.25; 61.51]
P1l-M1l	26.71 [12.33; 41.09]	27.63 [7.99; 47.27]	21.66 [9.78; 33.54]	17.78 [9.38; 26.18]	23.47 [13.28; 33.66]	23.40 [11.97; 34.83]	26.10 [14.49; 37.71]	31.54 [17.70; 45.38]

M1r, right first molar; P1r, right first premolar; I2r, right lateral incisor; I2l, left lateral incisor; P1l, left first premolar; M1l, left first molar.

**Table 4:** Evaluation of angular trueness per group at each location (mean ± 95% CI) in degrees.

	Medit Scanner				Zirkonzahn Scanner			
Angular Distance	Coltene Silicone	Coltene Stone Casts	Zhermack Silicone	Zhermack Stone Casts	Coltene Silicone	Coltene Stone Casts	Zhermack Silicone	Zhermack Stone Casts
M1r-P1r	0.46 [0.38; 0.54]	0.54 [0.37; 0.72]	0.42 [0.27; 0.56]	0.59 [0.34;0.84]	0.53 [0.30; 0.76]	0.54 [0.36; 0.72]	0.48 [0.32; 0.65]	0.49 [0.29; 0.69]
M1r-I1r	0.13 [0.06; 0.20]	0.21 [0.03; 0.39]	0.24 [0.01; 0.47]	0.33 [0.17; 0.49]	0.29 [0.04; 0.54]	0.09 [0.04; 0.14]	0.19 [0.09; 0.29]	0.15 [0.07; 0.22]
M1r-I1l	0.14 [0.06; 0.22]	0.26 [0.14; 0.38]	0.20 [0.11; 0.29]	0.40 [0.23; 0.58]	0.27 [0.05; 0.49]	0.23 [0.12; 0.34]	0.32 [0.20; 0.43]	0.37 [0.23; 0.51]
M1r-P1l	0.18 [0.09; 0.28]	0.26 [0.14; 0.38]	0.25 [0.18; 0.33]	0.38 [0.19; 0.58]	0.23 [0.11; 0.35]	0.17 [0.09; 0.26]	0.24 [−0.01; 0.48]	0.26 [0.14; 0.38]
M1r-M1l	0.49 [−0.27; 1.25]	0.26 [0.16; 0.37]	0.25 [0.06; 0.44]	0.29 [0.13; 0.45]	0.30 [−0.04; 0.63]	0.23 [0.14; 0.33]	0.21 [0.05; 0.36]	0.24 [0.09; 0.39]
P1r-I1r	0.10 [0.04; 0.15]	0.21 [0.09; 0.33]	0.43 [0.10; 0.77]	0.40 [0.11; 0.70]	0.18 [0.07; 0.29]	0.16 [0.05; 0.26]	0.25 [0.11; 0.39]	0.22 [0.10; 0.33]
P1r-I1l	0.15 [0.05; 0.25]	0.30 [0.08; 0.51]	0.31 [0.05; 0.57]	0.32 [0.05; 0.58]	0.21 [0.09; 0.33]	0.19 [0.03; 0.36]	0.36 [0.23; 0.50]	0.30 [0.15; 0.45]
P1r-P1l	0.35 [0.24; 0.47]	0.45 [0.28; 0.63]	0.53 [0.19; 0.86]	0.56 [0.30; 0.81]	0.44 [0.14; 0.74]	0.48 [0.35; 0.60]	0.78 [0.29; 1.28]	0.43 [0.25; 0.62]
P1r-M1l	0.54 [−0.29; 1.37]	0.38 [0.28; 0.49]	0.47 [0.20; 0.74]	0.45 [0.19; 0.72]	0.28 [−0.05; 0.60]	0.37 [0.22; 0.52]	0.46 [0.20; 0.71]	0.41 [0.21; 0.60]
I1r-I1l	0.14 [0.06; 0.23]	0.27 [0.04; 0.50]	0.24 [0.06; 0.41]	0.40 [0.12; 0.67]	0.22 [0.12; 0.31]	0.22 [0.07; 0.36]	0.25 [0.12; 0.38]	0.37 [0.21; 0.53]
I1r-P1l	0.08 [0.04; 0.14]	0.26 [0.07; 0.45]	0.43 [0.13; 0.72]	0.25 [0.04; 0.47]	0.21 [−0.09; 0.51]	0.18 [0.04; 0.32]	0.44 [−0.28; 1.15]	0.22 [0.09; 0.35]
I1r-M1l	0.18 [0.05; 0.31]	0.29 [0.13; 0.45]	0.44 [0.18; 0.71]	0.39 [0.20; 0.57]	0.36 [0.03; 0.69]	0.25 [0.13; 0.37]	0.39 [0.27; 0.51]	0.28 [0.15; 0.42]
I1l-P1l	0.17 [0.05; 0.28]	0.24 [0.11; 0.38]	0.26 [0.06; 0.45]	0.40 [0.19; 0.61]	0.16 [0.06; 0.26]	0.19 [0.03; 0.36]	0.52 [−0.11; 1.15]	0.27 [0.12; 0.42]
I1l-M1l	0.32 [0.09; 0.56]	0.32 [0.17; 0.47]	0.27 [0.16; 0.38]	0.55 [0.41; 0.69]	0.29 [0.06; 0.51]	0.33 [0.20; 0.46]	0.52 [0.39; 0.65]	0.61 [0.44; 0.79]
P1l-M1l	0.55 [−0.06; 1.16]	0.30 [0.07; 0.53]	0.31 [0.18; 0.45]	0.32 [0.09; 0.55]	0.40 [0.27; 0.53]	0.19 [0.07; 0.31]	0.44 [−0.03; 0.91]	0.28 [0.10; 0.45]

M1r, right first molar; P1r, right first premolar; I2r, right lateral incisor; I2l, left lateral incisor; P1l, left first premolar; M1l, left first molar.

## Data Availability

The original contributions presented in the study are included in the article, further inquiries can be directed to the corresponding authors.
